# Comparison of femoral mechanics before and after internal fixation removal and the effect of sclerosis on femoral stress: a finite element analysis

**DOI:** 10.1186/s12891-022-05888-4

**Published:** 2022-10-22

**Authors:** Yang Liu, Wenjie Song, Haoran Liang, Chaoxin Li, Wenjie Niu, Huifeng Shao, Yuze Wang, Ziquan Yang, Pengcui Li, Xiaogang Wu, Yong He, Xiaochun Wei, Wangping Duan

**Affiliations:** 1 Department of Orthopaedics, Second Hospital of Shanxi Medical University, No. 382, Wuyi Road, Taiyuan, 030001 Shanxi China; 2 Shanxi Key Laboratory of Bone and Soft Tissue Injury Repair, Taiyuan, 030001 China; 3 Institute of Biomedical Engineering, College of Biomedical Engineering, Taiyuan University of Technology, Taiyuan, 030024 China; 4 School of Mechanical Engineering, Hangzhou Dianzi University, Hangzhou, 310018 China; 5 State Key Laboratory of Fluid Power and Mechatronic Systems, School of Mechanical Engineering, Zhejiang University, Hangzhou, 310027 China; 6 Key Laboratory of 3D Printing Process and Equipment of Zhejiang Province, School of Mechanical Engineering, Zhejiang University, Hangzhou, 310027 China

**Keywords:** Femoral neck fracture, Removal of internal fixation, Sclerotic cancellous bone, Cannulated screw, Finite element analysis

## Abstract

**Background:**

Femoral neck fractures are a common traumatic injury. The removal of the internal fixation remains controversial, especially in terms of mechanical stability. Moreover, collapsed necrosis of the femoral head continues to occur after fracture healing. We believe that sclerotic cancellous bone (SCB) formation around the screw is associated with femoral head necrosis. We aimed to compare mechanical features before and after implant removal and determine the effect of SCB formation on stress distribution.

**Methods:**

Cylindrical cancellous bone sections were collected from a relatively normal region and an SCB region of a necrotic femoral head, and their elastic moduli were measured. Four femoral finite element models were developed: a) femoral neck fracture healing with implants, b) fracture healing without implants, c) sclerosis around the screw with implants, and d) sclerosis around the screw without implants.

**Results:**

The maximum von Mises peak stresses of models a and b were 66.643 MPa and 63.76 MPa, respectively, and were concentrated in the upper lateral femur. The main stress was scattered at the lowest screw tail, femoral calcar region, and lateral femur shaft. Moreover, coronal plane strain throughout the screw paths near the femoral head in models a and b was mostly in the range of 1000–3000 με. The maximum stress concentrations in models c and d were located at the lower femoral head and reached 91.199 MPa and 78.019 MPa, respectively.

**Conclusions:**

The stresses in the sclerotic model around the cannulated screws are more concentrated on the femoral head than in the healing model without sclerotic bone. The overall stresses in the healing femoral neck fracture model were essentially unchanged before and after removal of the internal fixation.

**Supplementary Information:**

The online version contains supplementary material available at 10.1186/s12891-022-05888-4.

## Background

While femoral neck fracture is a common type of traumatic injury, it is rare in the non-elderly population, where it is usually caused by high-energy trauma such as traffic accidents [1, 2]. Despite this low prevalence in the young population, increasing rates of several fracture-related complications and inherent higher activity levels make osteonecrosis of the femoral head (ONFH) more relevant to the health of adolescents. This is evidenced by their higher reported incidence of nonunion and ONFH than those in elderly people [3]. Clinicians have, thus, been searching for more appropriate surgical methods [4]. Among these, three partially threaded cannulated screws in an inverted triangle is the common traditional internal fixation method for femoral neck fractures in young patients [5, 6].

**Fig. 1:**
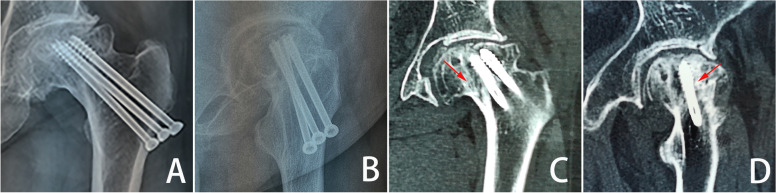
Radiographic data of a clinical femoral neck fracture case. **A** Coronal X-ray; **B** sagittal X-ray; **C** coronal CT; **D** sagittal CT. In the Figures, red arrows represent the high-density shadow around the cannulated screws

Following internal fixation of femoral neck fractures, and subsequentl fracture healing, the mechanical benefits of implants maintenance remain unclear. The rate of implant removal and the selection criteria for removal candidates are unknown, while the question of the implant’s post-healing effect on stress distribution remains controversial. Although both the femur and implants provide important mechanical effects, cases of postoperative femoral head collapse (Fig. 1) are still observed. Traditional cannulated screws are used as implant materials, and radiological examination of a collapsed necrotic femoral head sample 10 years after internal fixation showed numerous sclerotic and necrotic cavities formed around the implants (as shown in Fig. 1). We chose to use the term sclerotic cancellous bone (SCB) to describe this high-density distribution. Nevertheless, the longer the implants are retained, the more severe the osteosclerosis and osteonecrotic complications are likely to be, and the less likely it is that cancellous bone will develop normally.

Using three-dimensional finite element analysis (FEA), a previous study found evidence that subchondral bone may have little protective effect on the deep cancellous bone, which plays a major role in the degenerative collapse of the femoral head [7]. During bone reconstruction, both the proximal cortical and cancellous bones of the human femur have been found to form an optimal shape structure, with a balanced distribution of stress [8]. Living bone changes its composition, structure, and external morphology depending on the stress experienced. Experiments with controlled mechanical loads have provided a wealth of information on such functional adaptation. Cresswell et al. [9] demonstrated that bone formation within the first week after a mechanical stimulus is spatially correlated with areas of increased tissue strain/stress within cancellous bone. In a study of cancellous bone, in vivo loading led to an increase in bone volume fraction and density, and local mechanical strain and stress associated with long-term loading were demonstrated to predict the location of new bone formation to some degree [10]. Therefore, we think it is necessary to investigate the structure of bone trabecula in different regions of the proximal femur after sclerosis of the region around the screw paths, in order to explore the process of necrosis and the mechanism of femoral head collapse.

Femoral neck fractures accompanied by the collapse and ONFH are commonly seen in the elderly population, with high incidence of both within 3–5 years of healing, often requiring total hip arthroplasty. Although there is a gradual increase in the rate of femoral neck fracture healing among younger patients, the rising incidence of postoperative ONFH and collapse, which may be associated with higher activity levels [11], cannot be neglected. Since the protheses of patients have limited longevity, patients will likely require a revision surgery, which may lead to additional health implications for young patients. Thus, research on the mechanism of collapse and ONFH has become essential.

FEA is one of the most crucial tools for stress analysis in biomechanics research. Femoral head specimens are mostly derived from collapsed femoral heads removed during total hip arthroplasty in elderly patients. The structure of the test samples might be destroyed by real compression experiments, leading to poor reproducibility and difficulty in constructing a horizontal comparison. Therefore, this test applies linear FEA to simulate the compression test on the same cancellous bone specimens using different mechanical stimuli to observe the stress and strain distributions.

We hypothesize that the internal fixation technique has no decisive mechanical role after the femoral neck fracture has healed. Given that postoperative femoral head collapse nevertheless occurs, we further hypothesized that osteosclerosis leads to more stress being concentrated on the femoral head, eventually resulting in collapse and ONFH. By using FEA, we aimed to establish a model of sclerosis around the screw to investigate this assumption.

The objectives of this study were: first, to analyze the effect of implants on femoral stress and strain distribution after femoral neck fracture healing; and second, to determine the relationship between osteosclerosis around the screws and stress distribution in the proximal femur.

## Methods

The femoral heads were obtained from patients in our hospital with non-traumatic femoral head necrosis who underwent total hip arthroplasty, and those with femoral neck fractures. The study was approved by the Ethics Committee of the Second Hospital of Shanxi Medical University, and written informed consent was obtained from all participants.

**Fig. 2:**
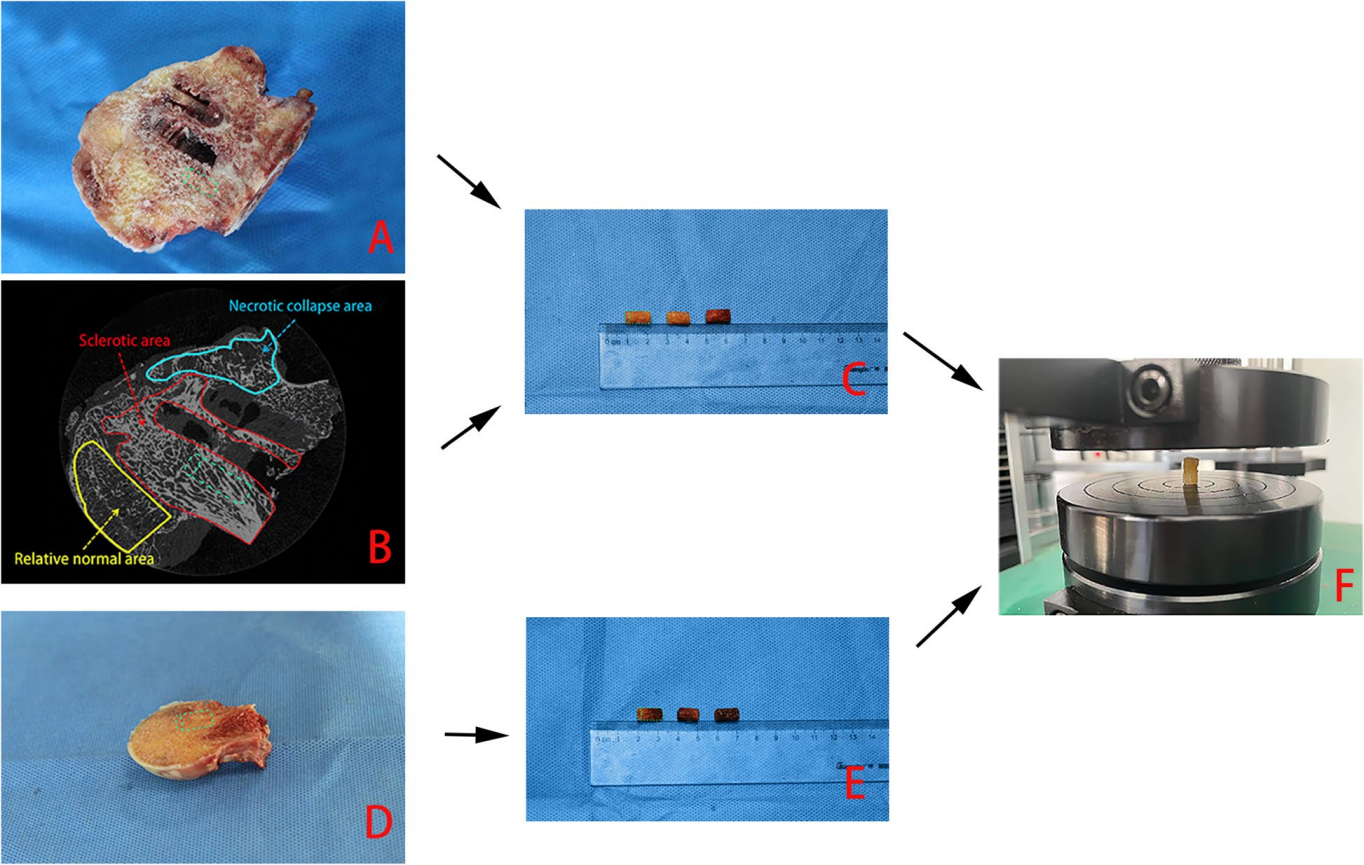
Measurement of the elastic modulus of cancellous bone associated with the sclerotic model. **A** The specimen of necrotic femoral head after internal fixation; **B** Micro-CT image of the specimen of necrotic femoral head; **C** cancellous bone cylinder as the elastic modulus of sclerotic region; **D** femoral head specimen after femoral neck fracture; **E** cancellous bone cylinder as the elastic modulus of relatively normal region; **F** universal testing machine to measure the elastic modulus of each cylinder

Since little information was available on the sclerotic region around the screws, we used micro-CT to assess the changes in bone microstructure in the different regions of femoral head specimens that had undergone osteonecrosis [12]. Samples were divided into necrotic collapse, sclerotic, and relatively healthy regions (as shown in Fig. 2B) [13]. The structural characteristics and arrangement of bone trabeculae differed significantly from region to region. In the sclerotic region, the bone trabeculae had a compact and regular arrangement, showed bone hyperplasia, and appeared thicker. Using a 6.5-mm diameter cartilage graft instrument, a cancellous bone cylinder 10 mm in length was removed from the region around the screw paths of the necrotic femoral head specimen (samples 1–6), and then a cylinder of the same size was removed from the femoral head specimen with a common femoral neck fracture (samples a-e). Subsequently, the elastic modulus of each sample was measured using a universal testing machine (model 1144, Instron; Norwood, MA) at the Taiyuan University of Technology (Fig. 2F). The mean elastic modulus of samples 1–6 was taken as the value of the sclerotic region and that of samples a-e was considered as the value of the relatively normal region.

**Fig. 3:**
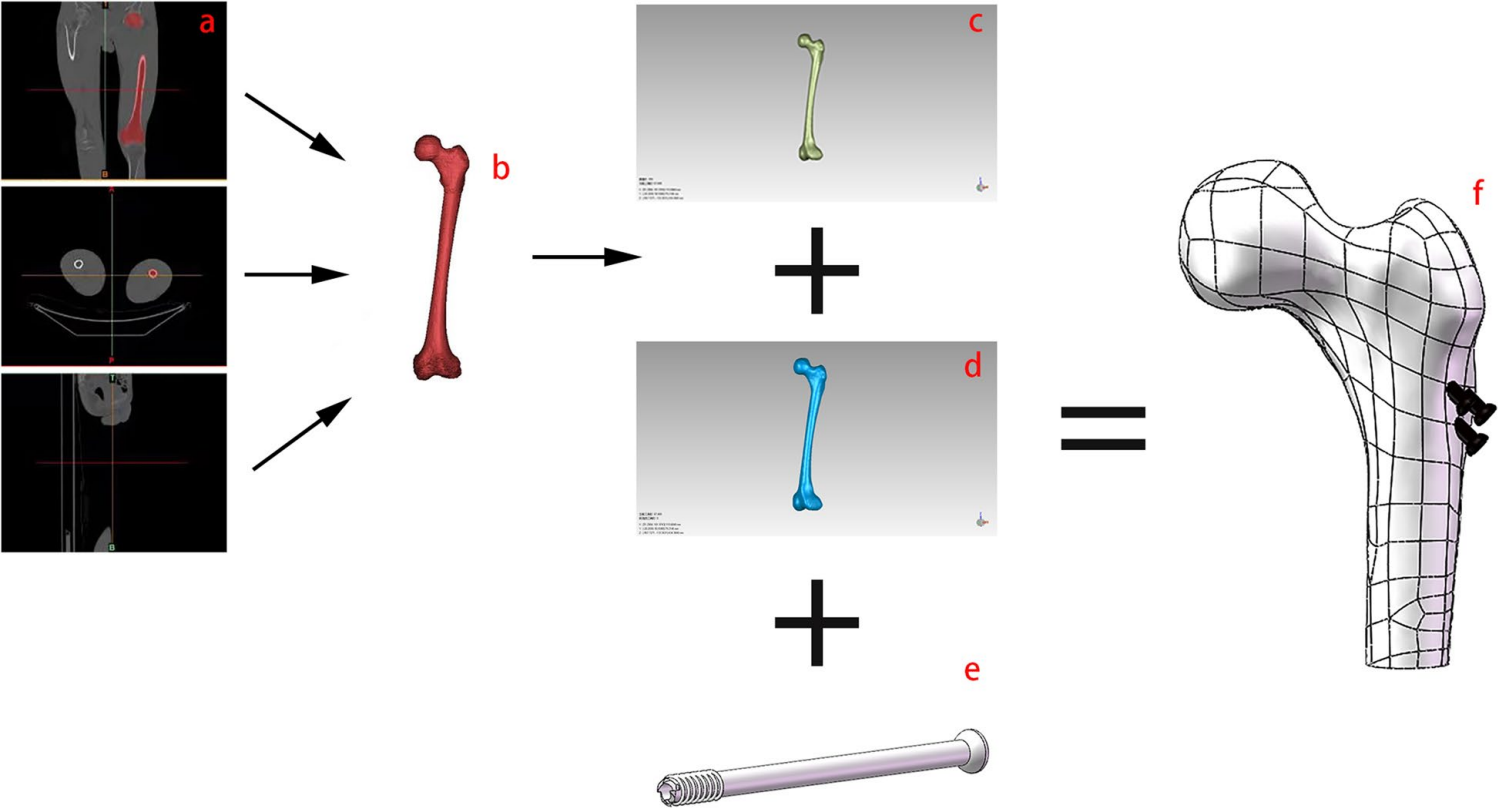
Constructing the finite element model with a normal femur. **a** Triple view of femoral CT; **b** rebuilt 3D model of femur; **c** cancellous bone after meshing, smoothing, and fitting surfaces; **d** cortical bone after meshing, smoothing, and fitting surfaces; **e** manufactured cannulated screw with a diameter of 7.3 mm; **f** reassembling the proximal femoral with screws

A healthy volunteer aged 30–35 years without any systemic disease or history of hip pathology was recruited. A CT scan of the femur with a layer thickness of 0.5 mm was performed using a GE Revolution CT scanner (GE Healthcare, Chicago, IL, USA) at 450 mA and 80 kV. The CT images were stored in DICOM format files and analyzed using the medical 3D reconstruction software Mimics 21 (Materialise, Leuven, Belgium) (Fig. 3a). A 3D model of the femur was built based on the gray value of the tissue and segmentation of the region (Fig. 3b), and the cortical bone and cancellous bone were preliminarily separated. The model was wrapped and smoothed to fill in holes and smoothen the surfaces, and the corresponding initial 3D mesh model was built (Fig. 3c d). The model was exported as an STL format file and incorporated into the Geomagic-Studio 2017 software (3D System Inc., Rock Hill, SC, USA) for meshing, smoothing, and fitting surface processing. After generating the surface sheets and fitting the surfaces, the completed surface models were exported into Solidworks 2017 (Solidwords Corp., Waltham, MA, USA).

Cannulated screws with a diameter of 7.3 mm were manufactured using Solidworks software according to the clinical data (Fig. 3e). Implants were arranged in a parallel fashion in an inverted triangle arrangement in the Solidworks software, according to a previously reported surgical method [14]. The angle of fixation was 135° with the longitudinal axis of the femur. Finally, the proximal femoral bone model was re-assembled (Fig. 3f). After all the models were prepared, FEA was performed using ANSYS17.0.

**Fig. 4:**
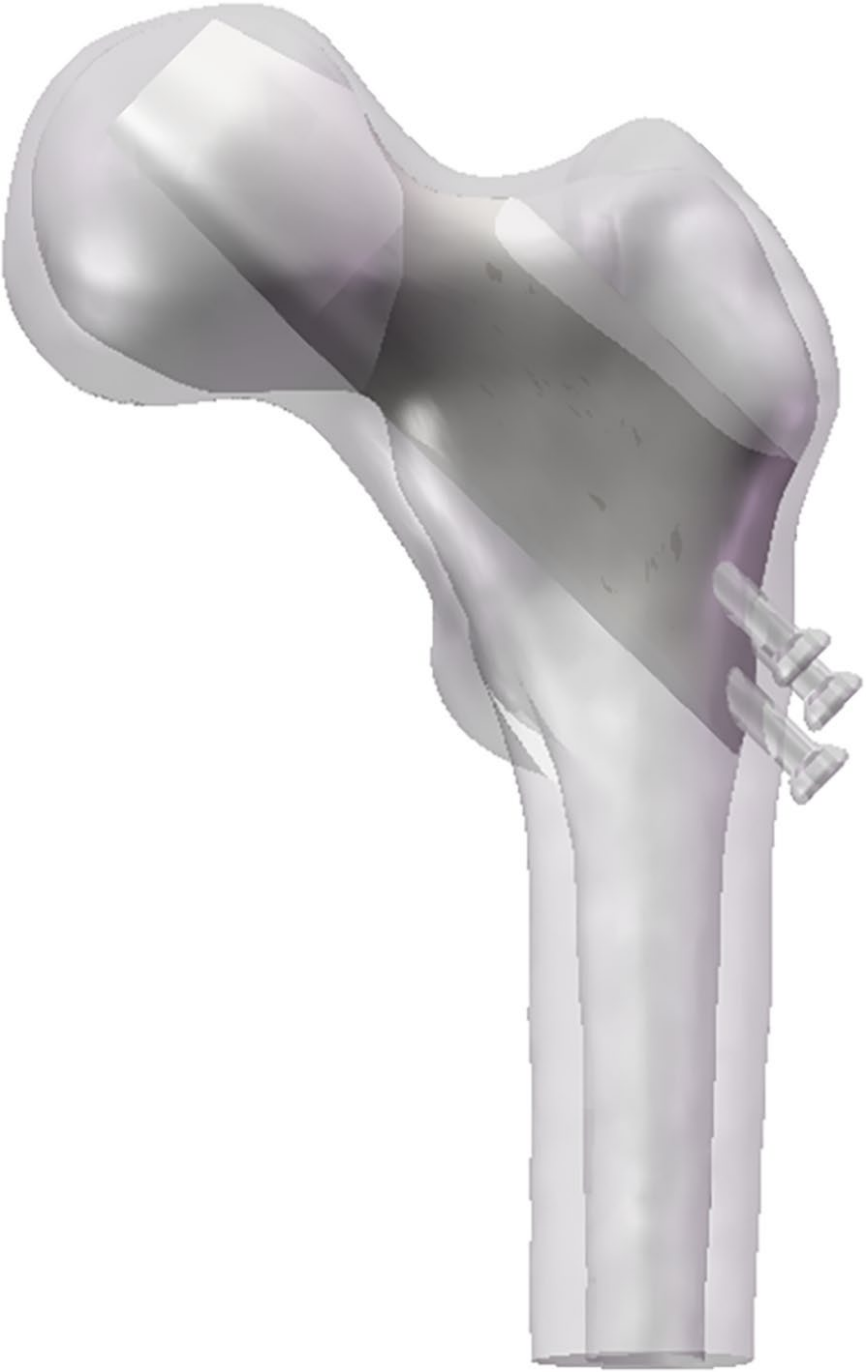
Proximal sclerotic femur model

Based on the micro-CT images (Fig. 2B), we concluded that the entire femoral neck was sclerosed. Therefore, the model of sclerosis around the screw was designed to extend the sclerotic region of the femoral neck upward in a frustum of a cone, beyond the cannulated screws and downward to the lateral femur. Meanwhile, the sclerotic bone filled the entire femoral neck (as shown in Fig. 4).

**Fig. 5:**
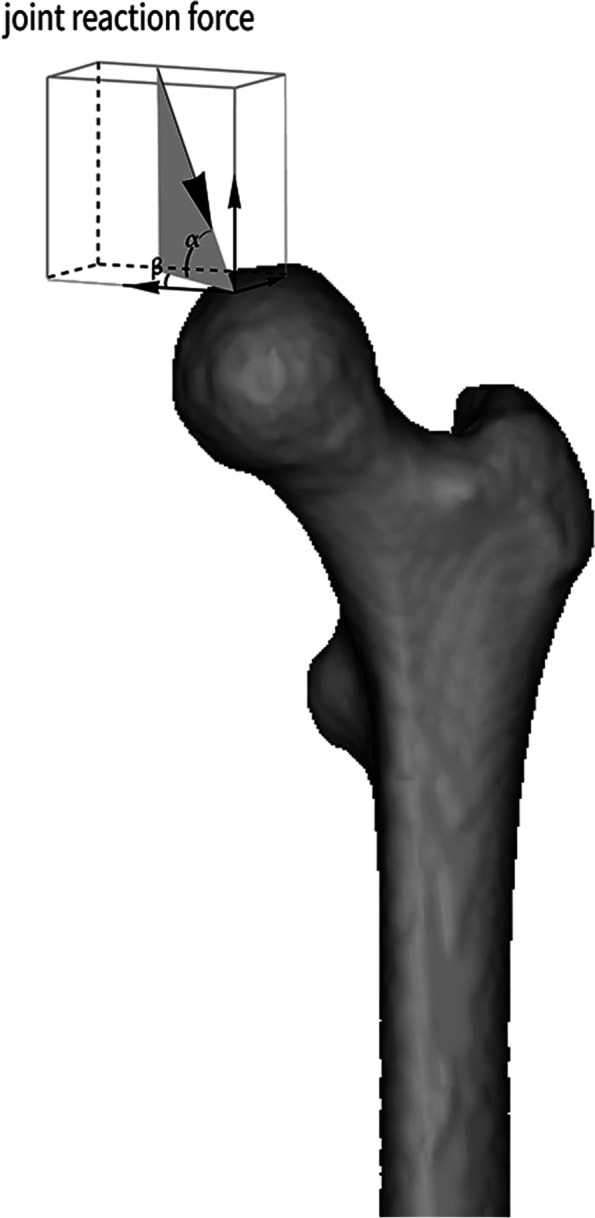
Setting the joint reaction force

The materials of the bone and screw in the model were assumed to be isotropic, homogeneous, and linearly elastic. Since the femur was taken from an older specimen, osteoporosis caused a relatively low elastic modulus of the cancellous bone. Accordingly, the elastic modulus of the cortical bone for the sclerotic model was chosen as a relatively low value, corresponding to that of elderly patients. For the fracture healing model, the elastic modulus of a healthy young person was chosen to correspond to the best mechanical conditions. The elastic modulus and Poisson’s ratio of various structural materials are listed in Table 1 [15]. The relationship between the thread and the cancellous bone was set to bound, while the relationship between the femoral bone and the rest of the cannulated screws was set as friction, with a coefficient of friction of 0.2. In a study by Van Houcke et al., a force corresponding to that of standing on one leg was applied to the corresponding cartilage surface of the femoral head, producing a joint reaction force of 1800 N, δ 159° and γ 7° (Fig. 5) [16]. To prevent rigid body motion, all nodes on the distal femur were constrained with zero degrees of freedom during the analysis. Due to the complexity of the finite element model, it was difficult to use hexahedral elements; thus, tetrahedral elements were employed in this study. Four femoral finite element models were developed: a) healing of the femoral neck fracture with implants, b) the same healing model without implants, c) sclerosis around the screw with implants, and d) sclerosis around the screw without implants. Mesh convergence studies showed that when the number of femur element is increased to 276,579, the incremental displacement of the model as the mesh changes is less than 4% and the change in displacement is almost negligible. The fracture healing models were discretized into 276,579 elements with 433,357 nodes and the sclerotic models were discretized into 163,233 elements with 272,524 nodes. Von Mises stress and strain were recorded.

**Table 1:** Material properties of the various components in the models

Item	Elastic modulus (MPa)	Poisson's ratio
Cortical bone of healing model	16800	0.3
Cancellous bone of healing model	840	0.2
Cortical bone of sclerotic model	13300	0.3
Sclerotic cancellous bone	240.07±33.40	0.2
Relatively normal cancellous bone	80.70±8.57	0.2
Cannulated screw	20600	0.3

## Results

### Measurements of the elastic modulus of cancellous bone

An examination of the interaction between abnormal bone architecture and in vivo injury in cancellous bone may offer insight into which architectural features are associated with possible complications of fractures and injury accumulation. The results of the elastic modulus of each sample are listed in Table 2. The elastic modulus of the sclerotic zone was measured at 240.07 ± 33.40 MPa, and that of normal cancellous bone was measured at 80.70 ± 8.57 MPa. The elastic modulus of the sclerotic region is three times higher than that of the normal cancellous bone, which is in line with our hypothesis. Moreover, this value is within the range of cancellous bone elastic moduli determined in other studies [17, 18] .

**Table 2:** Various elastic modulus from the necrotic femoral head specimen and the femoral head specimen in a common femoral neck fracture

	Diameter (mm)	Height (mm)	Elastic modulus (MPa)
Sample 1	6.40	10.84	216.87
Sample 2	6.02	10.80	187.36
Sample 3	5.98	8.86	238.21
Sample 4	6.34	10.07	392.28
Sample 5	6.12	12.36	248.76
Sample 6	5.85	12.52	156.95
Sample a	6.08	11.08	63.93
Sample b	6.23	9.55	96.87
Sample c	6.02	10.75	56.67
Sample d	5.99	10.51	97.84
Sample e	5.97	12.05	88.21

### Healing femur group

**Fig. 6:**
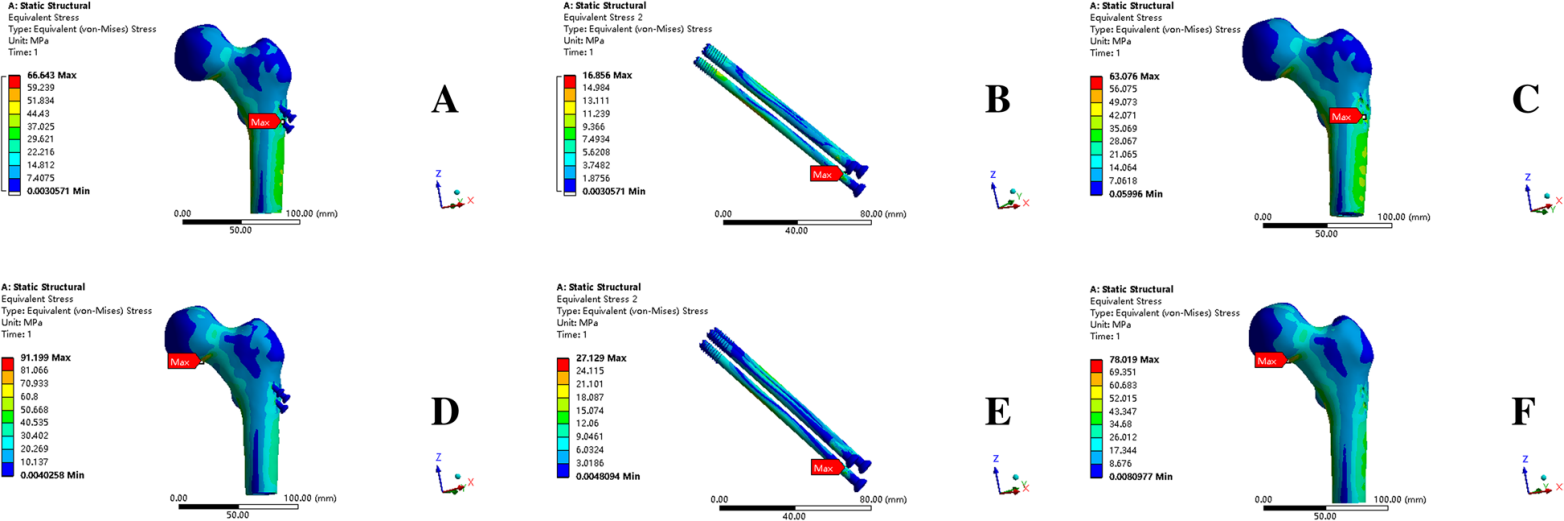
Stress distribution findings. **A** stress distribution of model a (the femoral neck fracture healing model with IFs); **B** the stress distribution of the IFs in model a; **C** stress distribution of the model b (the healing model); **D** stress distribution of model c (sclerosis around screws model with IFs); **E** stress distribution of the IFs in model c; **F** stress distribution of model d (sclerosis around screws model). IF, internal fixation

**Fig. 7:**
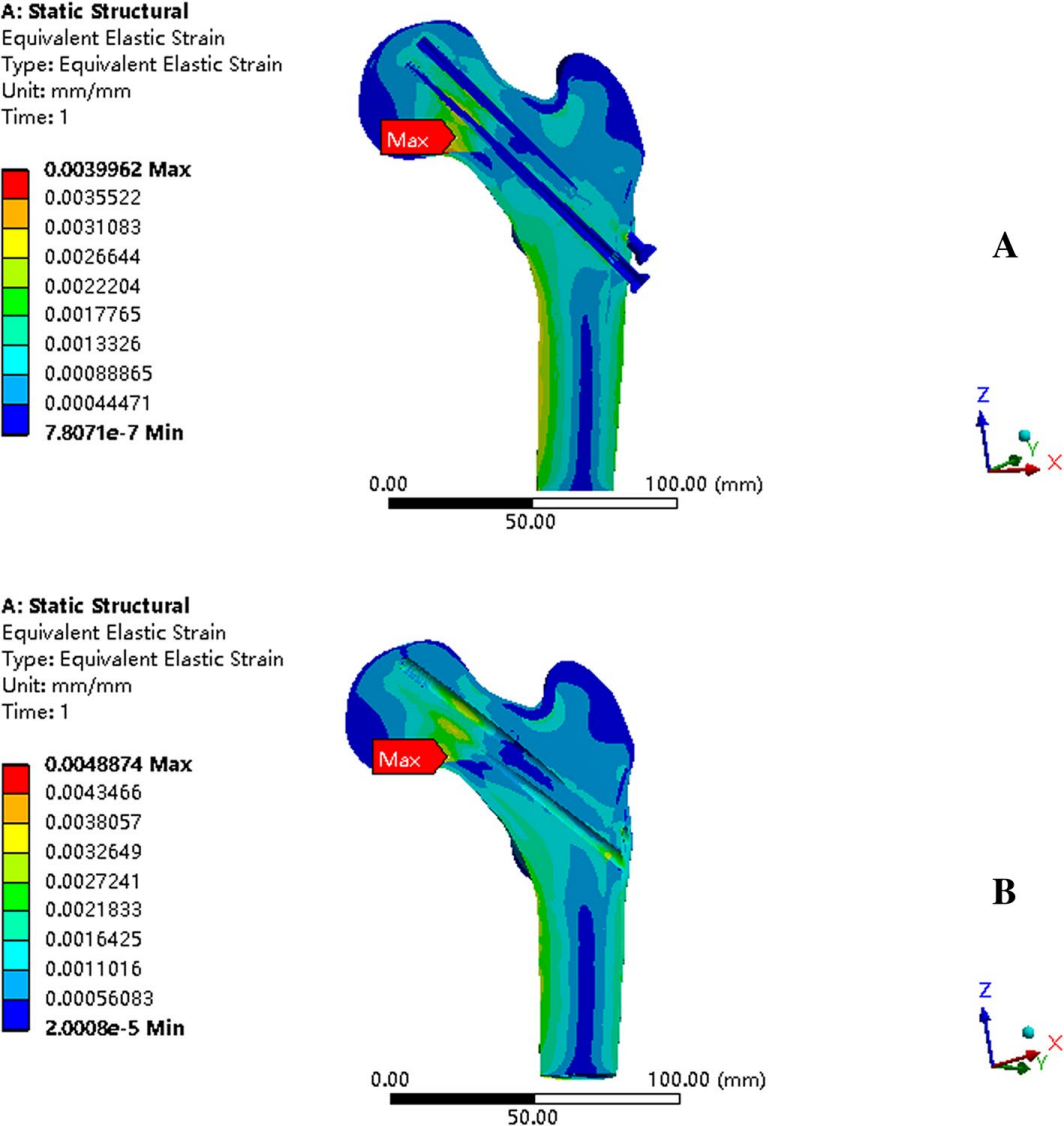
Strain distribution findings. **A** strain distribution in the coronal plane in model a; **B** strain distribution in the coronal plane in model b

In the a and b models (femoral neck fracture healing model with and without implants,respectively), the greatest stress concentration was scattered among the lowest screw tail, femoral calcar region, and lateral femur shaft. The maximum von Mises stresses in the two models were 66.643 MPa and 63.76 MPa, respectively, and the stress distributions in both models were generally consistent (Fig. 6A, C). The implants only partially absorbed the stress, resulting in a maximum von Mises stress of only 16.856 MPa (Fig. 6B). Therefore, the removal of implant does not significantly affect stress transfer during a static single-leg stance. Most of the coronal surface strains in models a and b were between 1000 and 3000 με and were located near the femoral head, where these sites are in a responsive osteogenic phase. In addition, the maximum strain for both models did not exceed 5000 με, indicating that cancellous bone is not at risk of fracture during static standing (Fig. 7).

### Sclerotic femur group

The maximum von Mises stress concentrations in the c and d models (sclerosis around the screws with and without implants, respectively) were in the lower femoral head, at 91.199 MPa and 78.019 MPa, respectively (Fig. 6D, F). The presence of sclerotic cancellous bone concentrates stress on the top of the femoral head, increasing the chances of femoral head necrosis. In addition, the other regions of stress dispersion were consistent with the results of the a and b models. The implants had a maximum von Mises stress of 27.129 MPa (Fig. 6E), and their presence did not affect the location of stress concentration.

## Discussion

Some researchers believe that internal fixations cannot be removed [19]. Patients with an evident implant back-out on X-ray and Pauwels type III fracture require implant removal due to discomfort. Implant removal is more likely to affect the blood supply to the femoral head and contribute to femoral head necrosis after bone healing. Moreover, some surgical procedures can lead to blood accumulation in the joint, resulting in increased intracapsular pressure. Surgery can stimulate local vasospasm, leaving the blood in a hypercoagulable state, which can easily lead to the formation of a local thrombus, further reducing the blood supply to the femoral head and causing femoral head necrosis [20]. Due to the effect of daily activities, microfractures of the femoral head, which are associated with decreased bone density, may occur after the removal of the cannulated screws [21]. In contrast, some medical professionals do believe that internal fixation can be removed. Using a multivariate logistic regression analysis, Wang et al. found that implant removal was not a significant risk factor for femoral head necrosis [22]. Implant removal appears to be a safe procedure with little risk, and none of the adverse events that occurred were serious or resulted in permanent disability. The significant improvement in blood supply to the femoral head is due to a decrease in intraosseous pressure. When the implants are removed, the intraosseous pressure in the femoral head is reduced, which facilitates the recovery of blood flow. Zielinski et al. [23] found that implant removal after internal fixation of a femoral neck fracture had a significant positive effect on patient function. Even if a fracture heals well, implants in femoral neck fractures can lead to long-term pain and physical limitations. These limitations may be caused by scarring, tissue damage, femoral neck shortening due to impact at the fracture site, and loss of muscle strength due to surgical exposure and injury. Weight bearing may cause impaction at the fracture site, which may result in interference with the surrounding soft tissues (i.e., fascia lata and abductor muscles) [23]. This can lead to functional impairment and local irritation [24]. The divergent opinions of various studies have led to an absence of uniform surgical guidelines for postoperative femoral neck fractures, especially in terms of mechanical findings. An increasing number of scholars emphasize that biomechanical factors play a significant role in the process of necrosis, while insufficient blood supply had not been found to explain the mechanism of necrosis. However, a subsequent study found that necrosis could also be observed in areas with an adequate blood supply. In fact, the stress concentration region of the femoral head in daily life was found to be the same region as that of femoral head necrosis and collapse [25]. From the results seen in section 3.2, it appears that the location of stress distribution was essentially the same for models a and b, with the implants absorbing some of the stress, leading to a maximum von Mises stress of only 16.856 MPa. Therefore, the removal of implant does not significantly affect stress transfer during a static single-leg stance. Mechanically, removal of the implant in a healed femoral neck fracture would therefore have no effect on stress concentration.

As found in the sclerotic femur group, the maximum von Mises stresses were concentrated at the lower part of the femoral head for models c and d. In addition, compared with the healing model, the stress at the top of the femoral head increased from approximately 10 MPa to approximately 30 MPa. It is suggested that the presence of sclerosis allowed the stress to be concentrated in the femoral head, preventing partial stress from being transmitted to the lateral femoral stem. For this reason, even if the femoral neck fracture heals well, the femoral head is still at risk of collapse as long as sclerosis is present. Therefore, finding ways to prevent the formation of sclerosis will be the next step in our research.

Bone resorption and formation were relatively balanced under normal physiological stress range (approximately 100–1000 με). Loading in the range of 1000 to 3000 με results in active formation of bone tissue, increased bone volume, and bone strength. Loads exceeding 3000 με lead to the accumulation of micro-damage in the bone tissue, which in turn reduces bone strength. In addition, there is a risk of fracture when the load exceeds 5000 με [26]. Excessive bone microdamage cannot be repaired completely; thus, this results in trabecular and fatigue fractures. In other words, exposing the trabeculae around the screw paths to strains of 1000 to 3000 με leaves these sites in a responsive osteogenic phase, resulting in the formation of sclerotic bone. The strain in both models did not exceed 5000 με, suggesting that the cancellous bone was not at risk of fracture during static standing, which also confirms that implant removal is plausible in terms of mechanics. The existence of implants contributed to lower strain to the entire cancellous bone but did not alter the general strain distribution.

Lin et al. [27], using FEA, found that for the screw axis to be as close to the negative gravity line as possible, the lower screw would have to be closer to the vertical angle. The conventional surgical position of the lower screw increases the stress concentration and does not transfer the body compressive stress along the screw to the external femoral stem cortex. Wang et al. [25] also using FEA, found that stress distribution at the proximal femur significantly changed with the angle of the femoral and spatial displacement after internal fixation. The calcar femoral region along with other structures work as a bearing system in accordance with the physiological requirements, to absorb shock and deflect impact load more effectively. Normally, the stressed cortical and cancellous bone tissue transmits stress to the trochanter, where the greatest femoral trabecular stress is concentrated. After internal fixation, the stress concentration is transferred to the upper part of the head–neck junction, so that the trabecular orientation no longer coincides with the forces applied to the femoral head. The trabecular bone of the femoral head is subjected to both axial and shear stress, which adversely affects the stability of the trabecular structure. The inconsistency between the location of the main pressure on trabecular bone and that of the stress region may led to adaptive remodeling [28]. Wang et al. [29] found that reducing mechanical load reduced the thickness of bone trabeculae and increased the volume fraction of rod trabeculae through the conversion of plate trabeculae by simulating trabecular 3D reconstruction. In addition, trabecular plates were more involved in bone remodeling and played a leading role in sustaining the external load. Therefore, trabeculae around the screw may be partially converted from rods into plates, concentrating and thickening to form plate sclerotic bone, to withstand a larger external load [12].

Zhang et al. found that the cortical bone of mature rats did not undergo bone construction under physiological conditions, whereas intracortical bone reconstruction occurred at sites of bone microdamage after axial forelimb loading. In contrast, this did not occur when the same load was applied but no bone microdamage was produced, suggesting that bone microdamage could initiate bone tissue reconstruction [15]. As shown in the strain results, bone tissue strains greater than 3000 με initiated the bone reconstruction. A characteristic bony structure found during revision surgery for failed arthroplasty, the sclerotic bone rim, is the continuous bone segment closest to the surface of the implant [30]. The loose cement mantle and implant are in an unstable mechanical environment that has the potential to produce thicker, more continuous, distinct,and sustained formative activity at both surfaces of the sclerotic rim. After internal fixation for femoral neck fracture, both the stress concentration around the cannulated screws and the apparent implant recession provide unstable conditions for the formation of sclerotic bone around the screw. The microdamage caused by the implants initiates bone reconstruction, and these unstable conditions allow for progressive thickening and expansion of the sclerosis.

Therefore, the formation mechanism of the sclerotic region is as follows: the lower screw of the normal inverted triangular internal fixation increases the stress concentration. The change in stress location following internal fixation then causes adaptive remodeling of cancellous bone, and the trabeculae around the internal fixation is transformed into plate-like trabeculae in order to take on greater stress. Microdamage around the implants initiates bone reconstruction and the easily displaced implants create unstable conditions for progressive thickening and expansion of the sclerosis. Finally, the formation of a high-density shadow is observed in the CT image, as seen in the presented case (Fig. 1).

The osteoclastic process requires only 3 weeks to alter the trabecular bone structure, whereas osteogenesis requires 3 months to construct new bone with good mechanical performance [13]. When the increase in bone mass does not compensate for the osteolysis caused by chronic high stress stimulation around the screws, bone reconstruction is obstructed, and this initiates the first step in the prevention of osteogenesis of screw paths. The implant may be considered as a foreign body to the bone marrow space, and the peri-implant fracture trabeculae at the drilled surface can separate the bone marrow space from the implant by forming a sclerotic rim in response to pressure. The presence of SCB also separates the screw paths from the bone marrow space and does not allow for the effective osteogenic component to pass through, which is one of the reasons why the screw paths cannot undergo bone reconstruction even after implants are removed.

This study demonstrates that removal of the implants does not affect stress concentrations and that the cannulated screw causes sclerosis around the screw. Therefore, the cannulated screw should be removed after the femoral neck fracture has healed.

The limitations of this study include the following: we assumed the existence of a linear elastic mechanical model of the proximal femur, and ignored the influence of ligaments and surrounding muscles on fracture stability, to simplify the internal fixation and the fracture model; whereas in fact, bone is an anisotropic material, and its elastic behavior can be best described as orthotropic [31]. Evidence indicates that fluid flow and pressure in the marrow space and fluid flow within the lacunar canalicular network are associated with bone formation, although stress and strain in mineralized tissue is believed to be the primary stimulus [32, 33]. Although increased mechanical stimulation leads to new bone formation, the mechanical conduction mechanisms are complex, and the exact mechanism remains unclear. More clinical trials and realistic biomechanical tests are needed to overcome the limitations of our study.

## Conclusion

In summary, the overall stresses in the healing femoral neck fracture model were essentially unchanged before and after removal of the internal fixation. After internal fixation, the cancellous bone around the implant was mostly in an osteogenic state. The stresses in the sclerotic model around the cannulated screws are more concentrated on the femoral head than in the healing model without sclerotic bone. This paper also discusses the mechanisms of sclerosis formation and the reasons for the non-growth of screw paths, and recommends removal of screws after fracture healing.

## Supplementary Information

**Additional file 1.**

**Additional file 2.**

## Data Availability

The datasets used or analyzed during the current study are available from the corresponding author on reasonable request.
