# Supplementary figures and images for: Comparison of femoral mechanics before and after internal fixation removal and the effect of sclerosis on femoral stress: a finite element analysis
Source: BMC Musculoskelet Disord. 2022 Oct 22;23:930. doi: 10.1186/s12891-022-05888-4 (PMC9587577; doi:10.1186/s12891-022-05888-4)

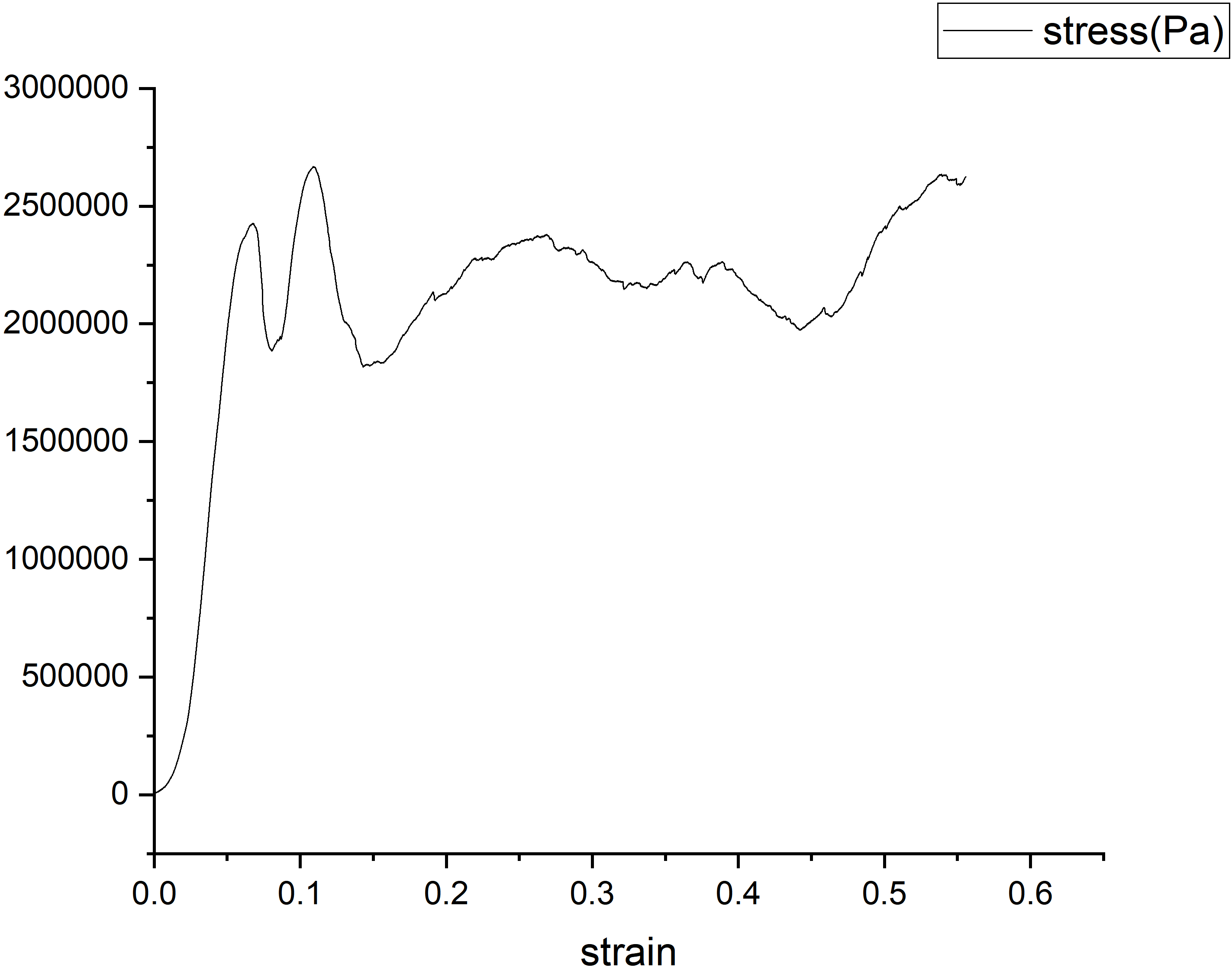

Supplement: Supplementary file 1 — Additional file 1. [file 12891_2022_5888_MOESM1_ESM.zip › Normal 1R5.tif]

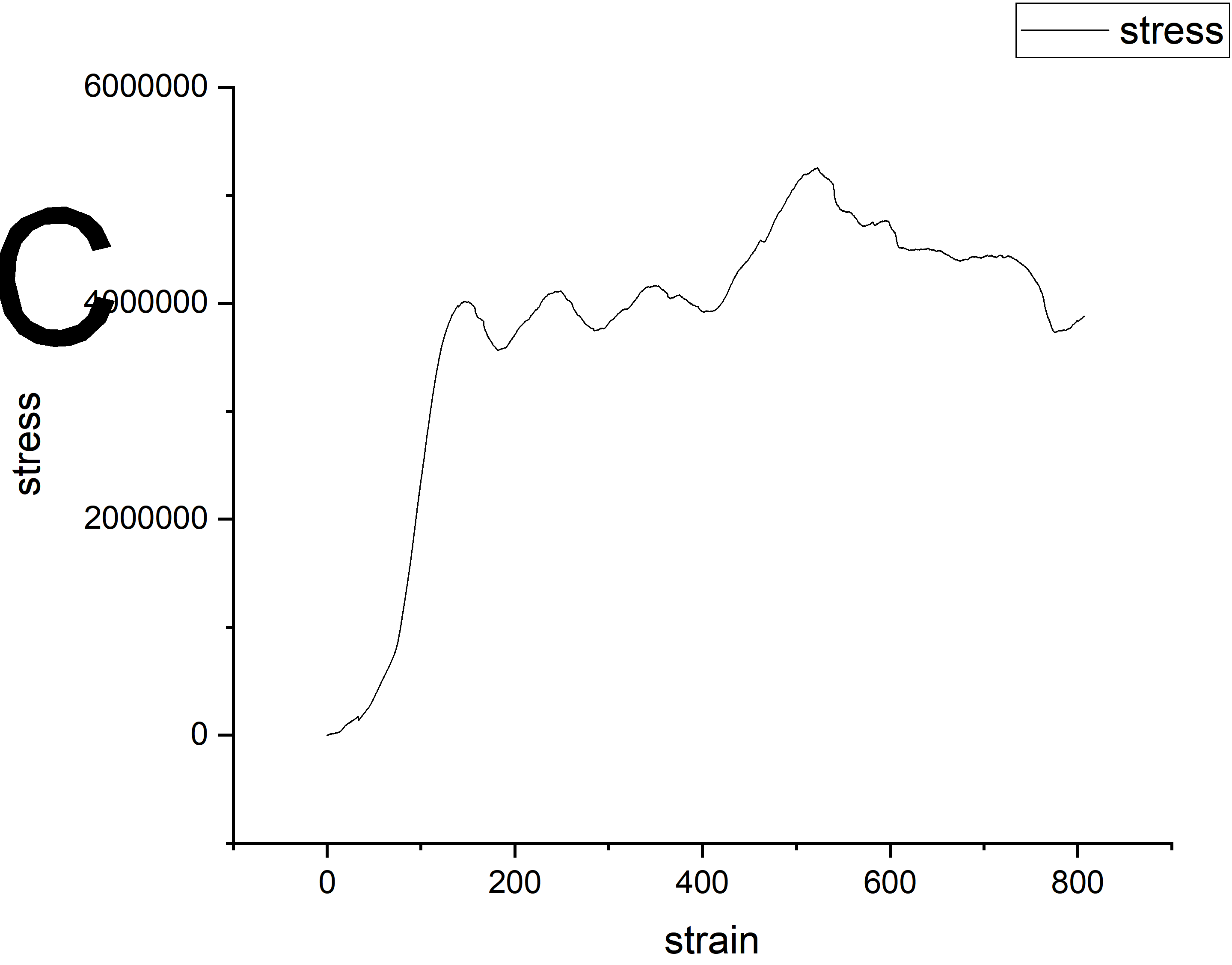

Supplement: Supplementary file 1 — Additional file 1. [file 12891_2022_5888_MOESM1_ESM.zip › Normal 2R5.tif]

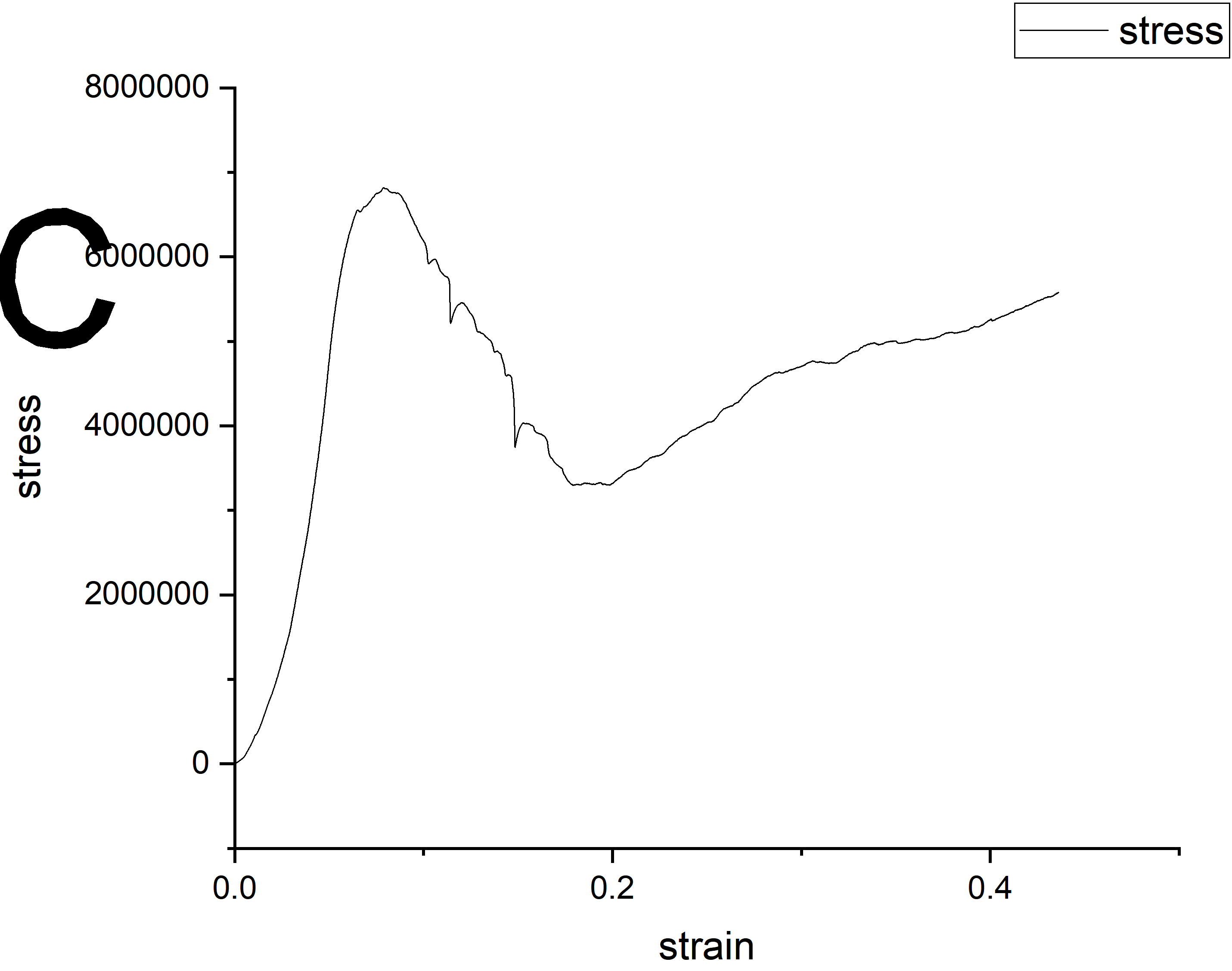

Supplement: Supplementary file 1 — Additional file 1. [file 12891_2022_5888_MOESM1_ESM.zip › Normal 3R5.tif]

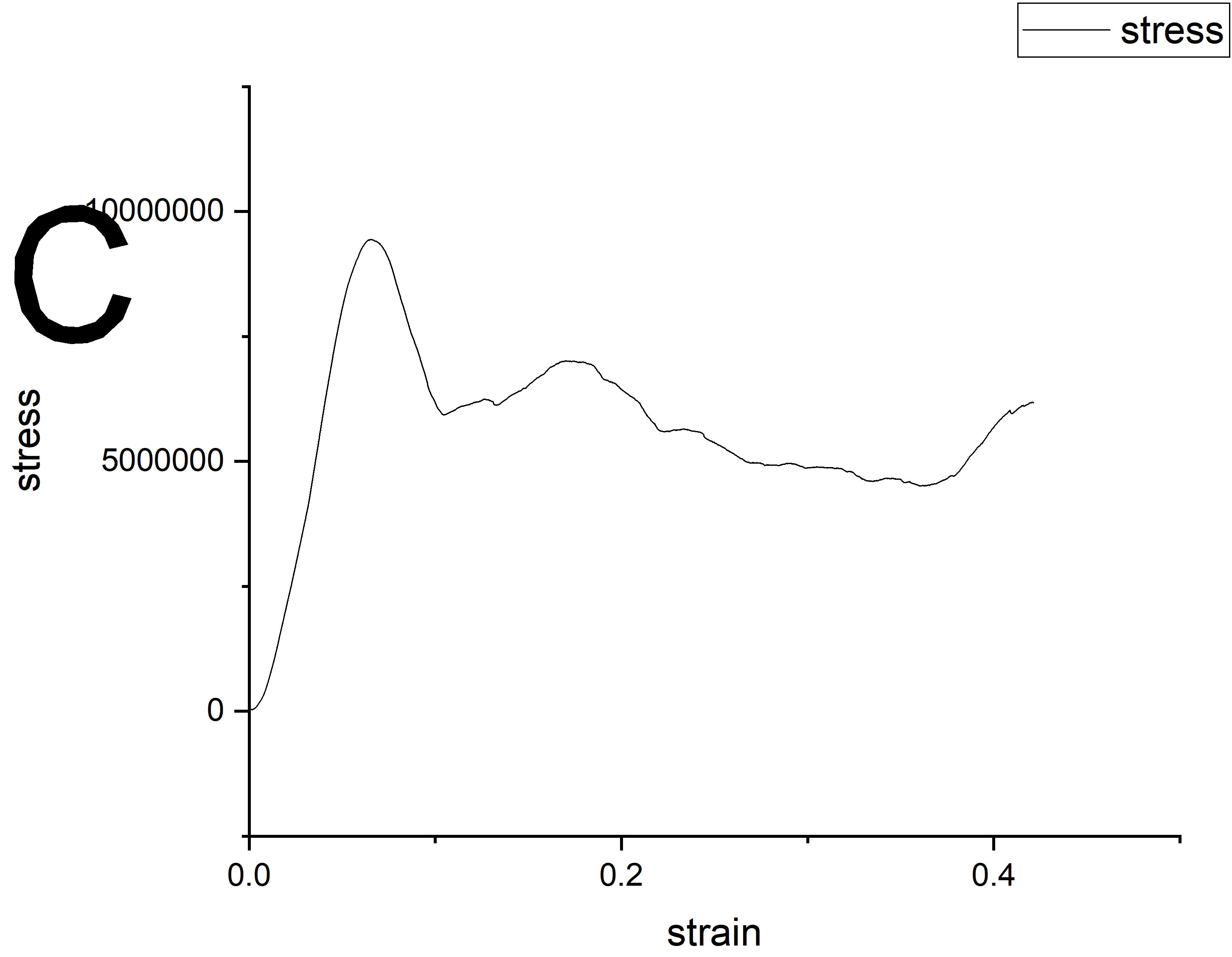

Supplement: Supplementary file 1 — Additional file 1. [file 12891_2022_5888_MOESM1_ESM.zip › Normal 4R5.tif]

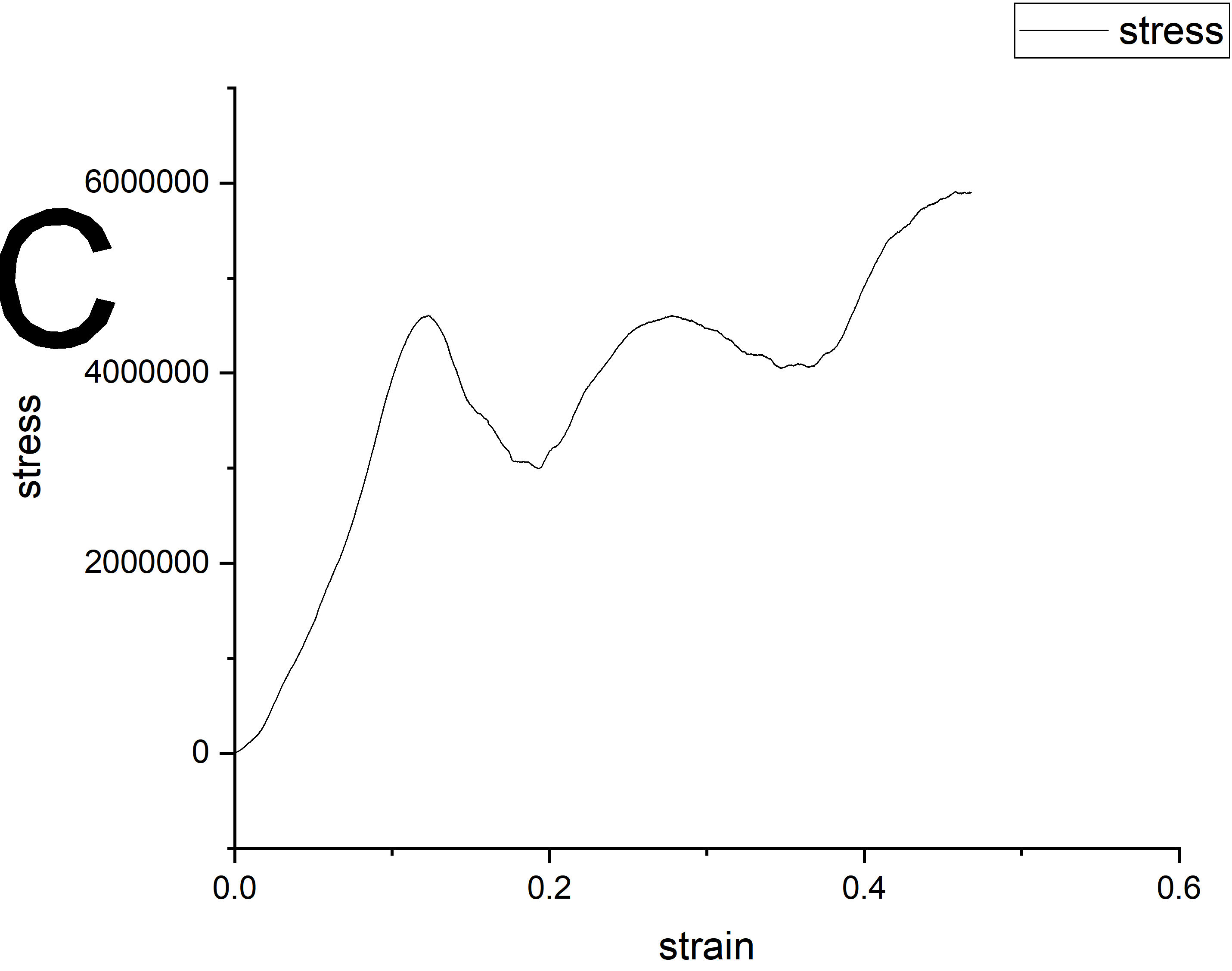

Supplement: Supplementary file 1 — Additional file 1. [file 12891_2022_5888_MOESM1_ESM.zip › Normal 5R5.tif]

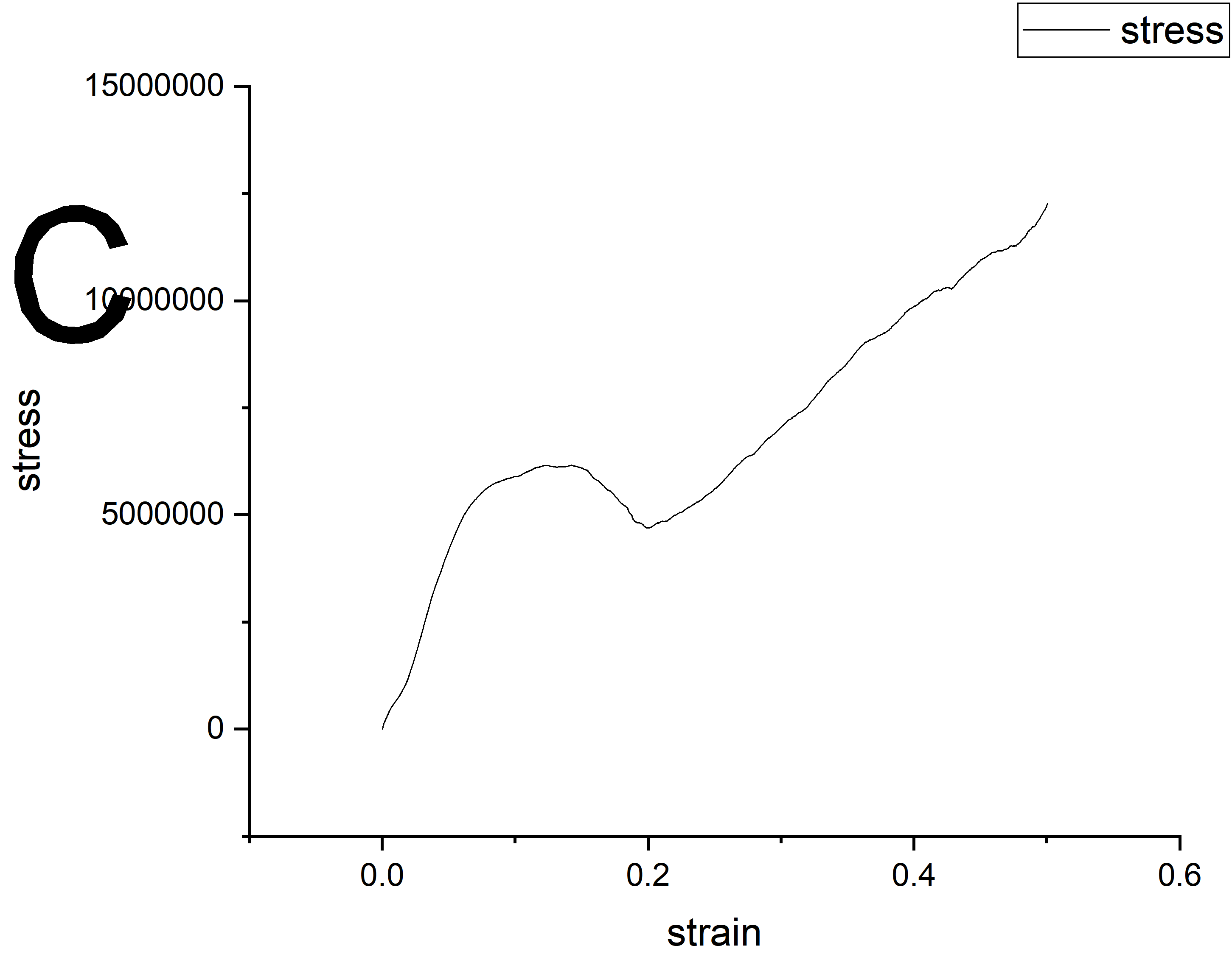

Supplement: Supplementary file 2 — Additional file 2. [file 12891_2022_5888_MOESM2_ESM.zip › Sclerosis1R5.tif]

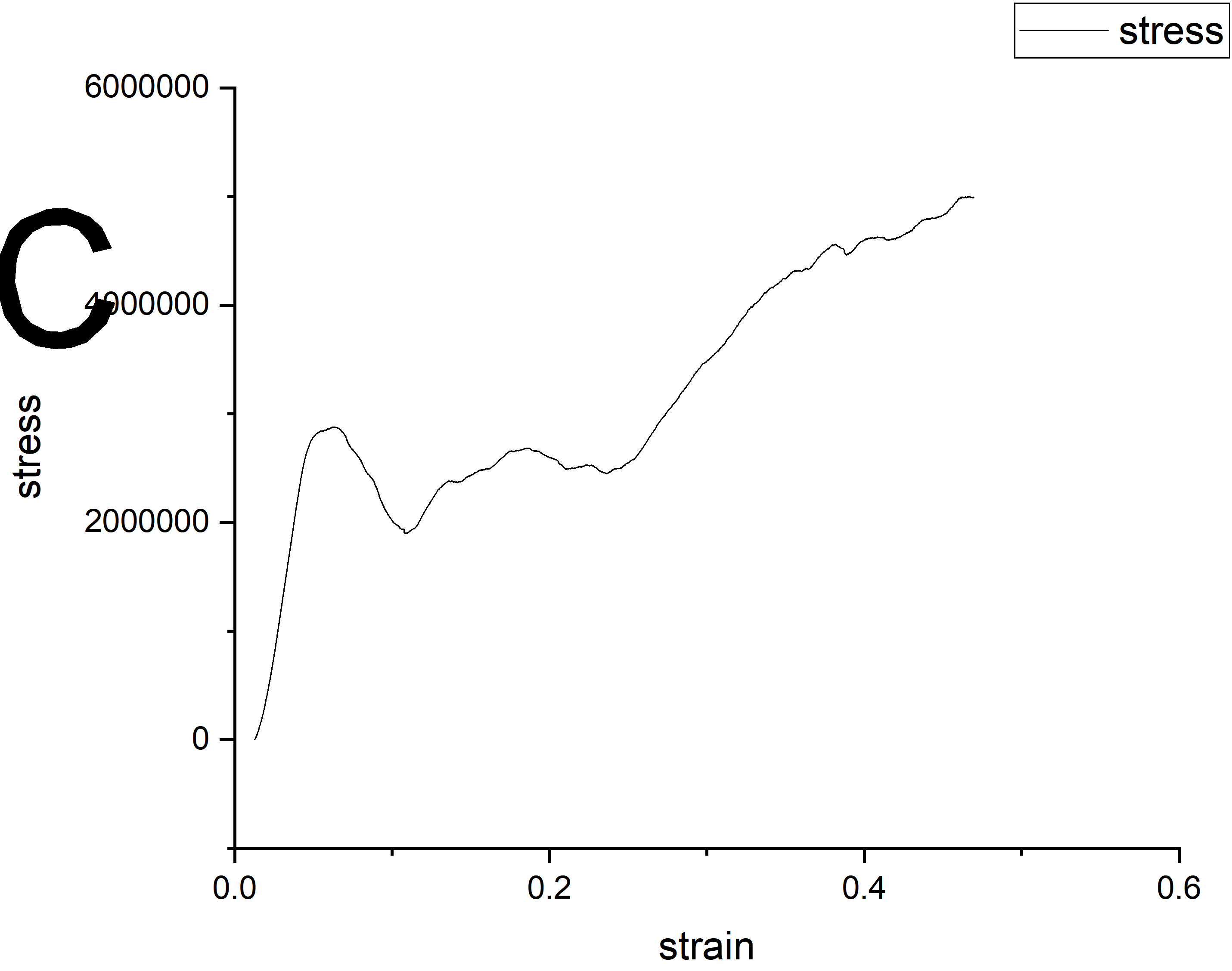

Supplement: Supplementary file 2 — Additional file 2. [file 12891_2022_5888_MOESM2_ESM.zip › sclerosis2R5.tif]

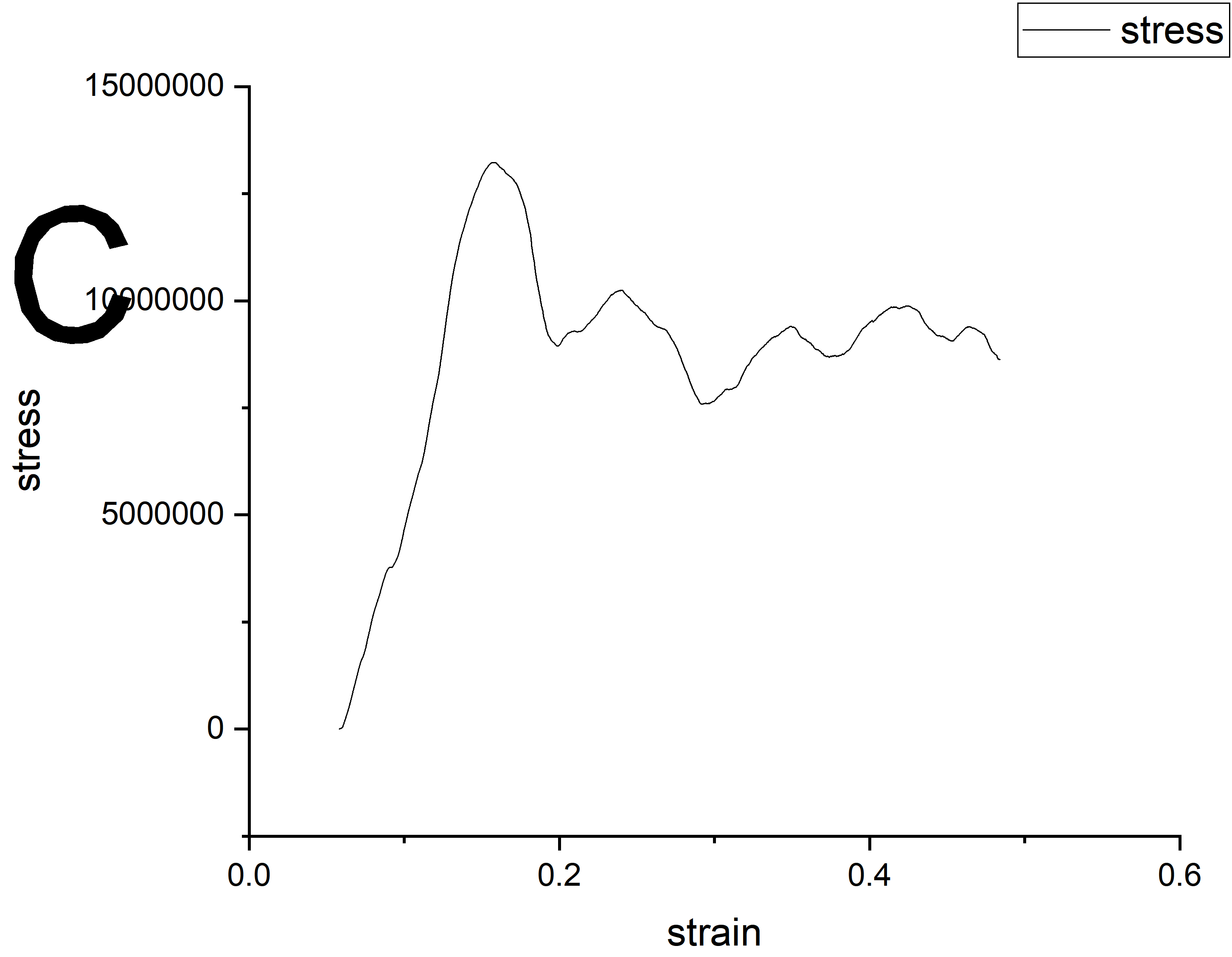

Supplement: Supplementary file 2 — Additional file 2. [file 12891_2022_5888_MOESM2_ESM.zip › sclerosis3R5.tif]

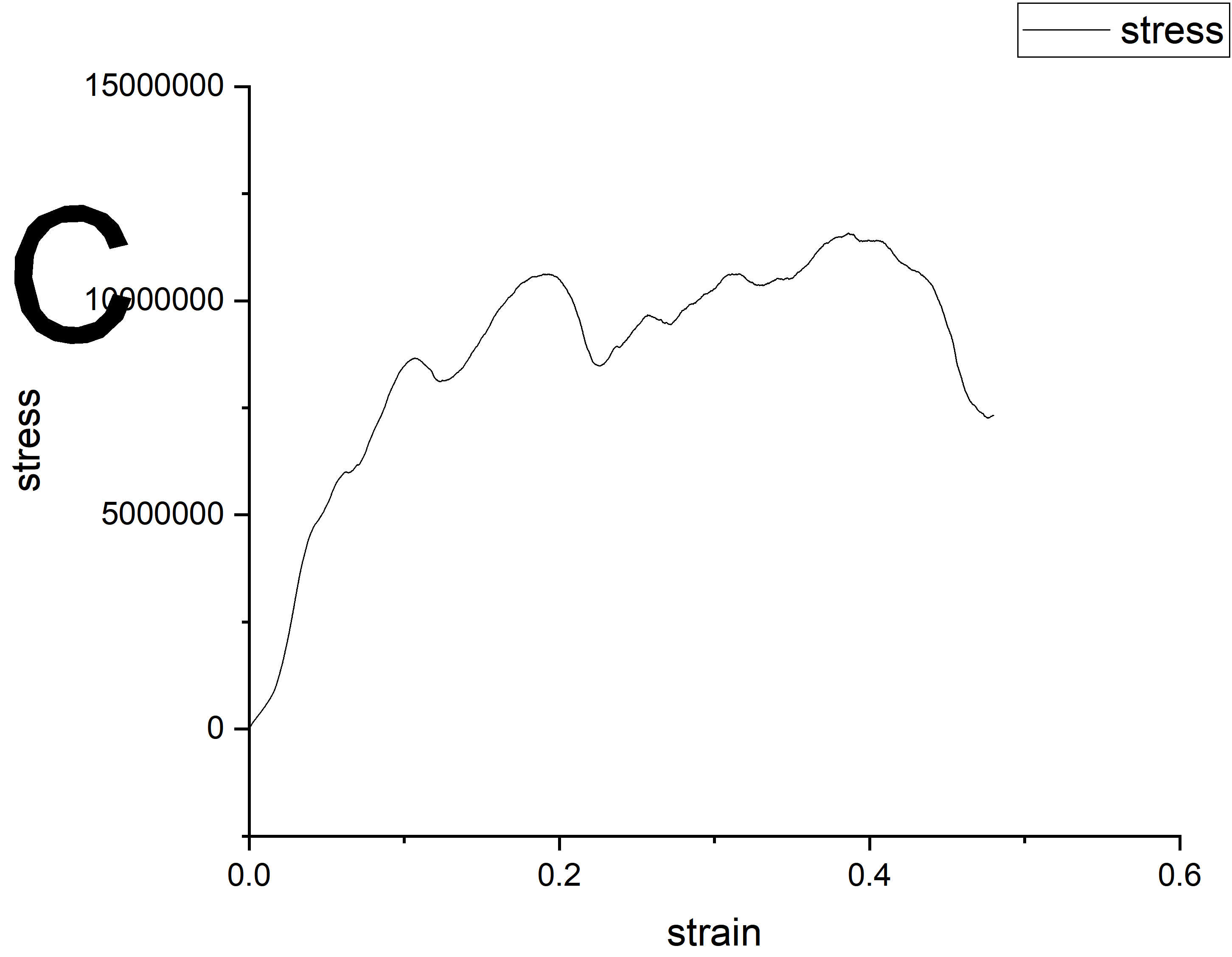

Supplement: Supplementary file 2 — Additional file 2. [file 12891_2022_5888_MOESM2_ESM.zip › sclerosis4R5.tif]

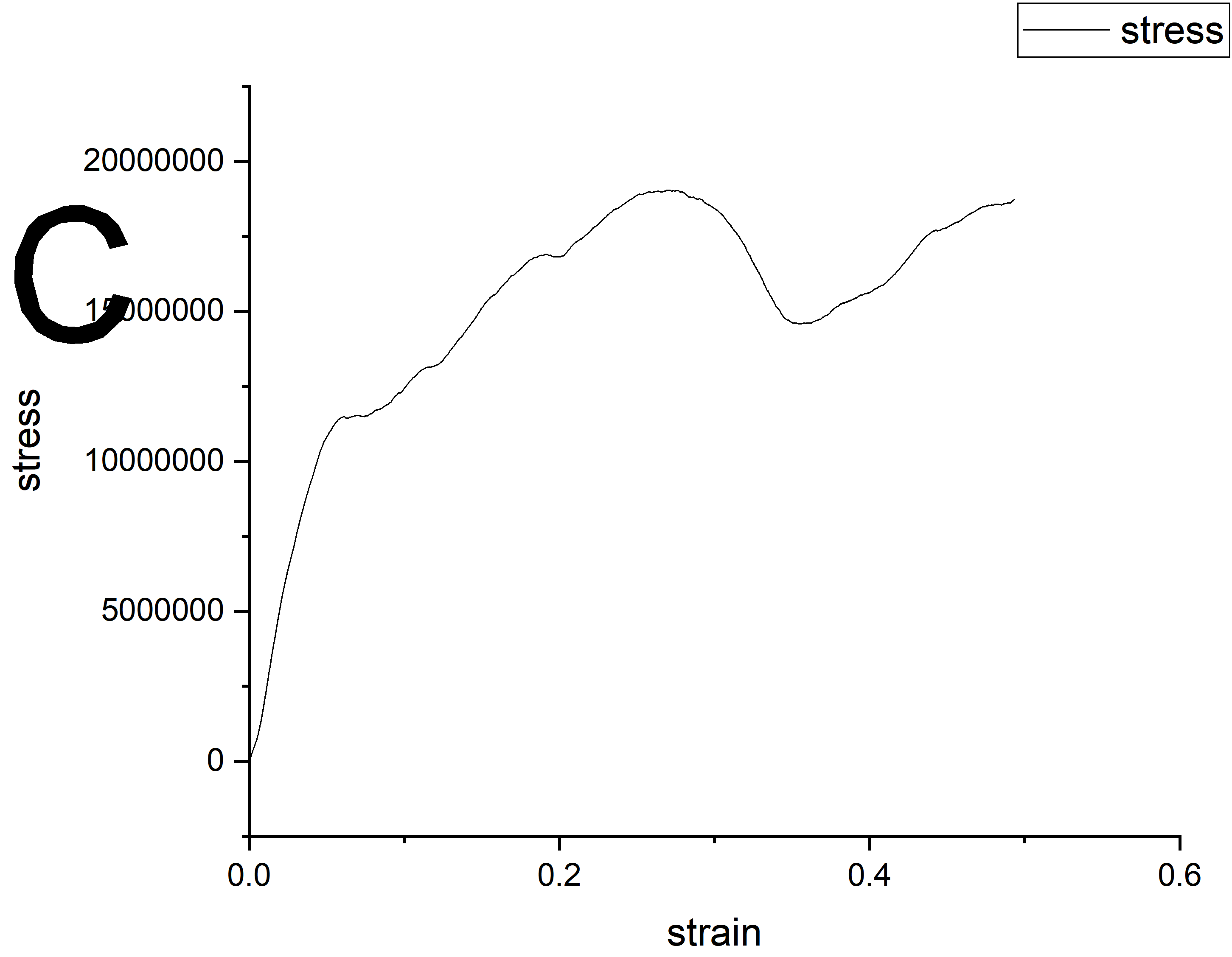

Supplement: Supplementary file 2 — Additional file 2. [file 12891_2022_5888_MOESM2_ESM.zip › sclerosis5R5.tif]

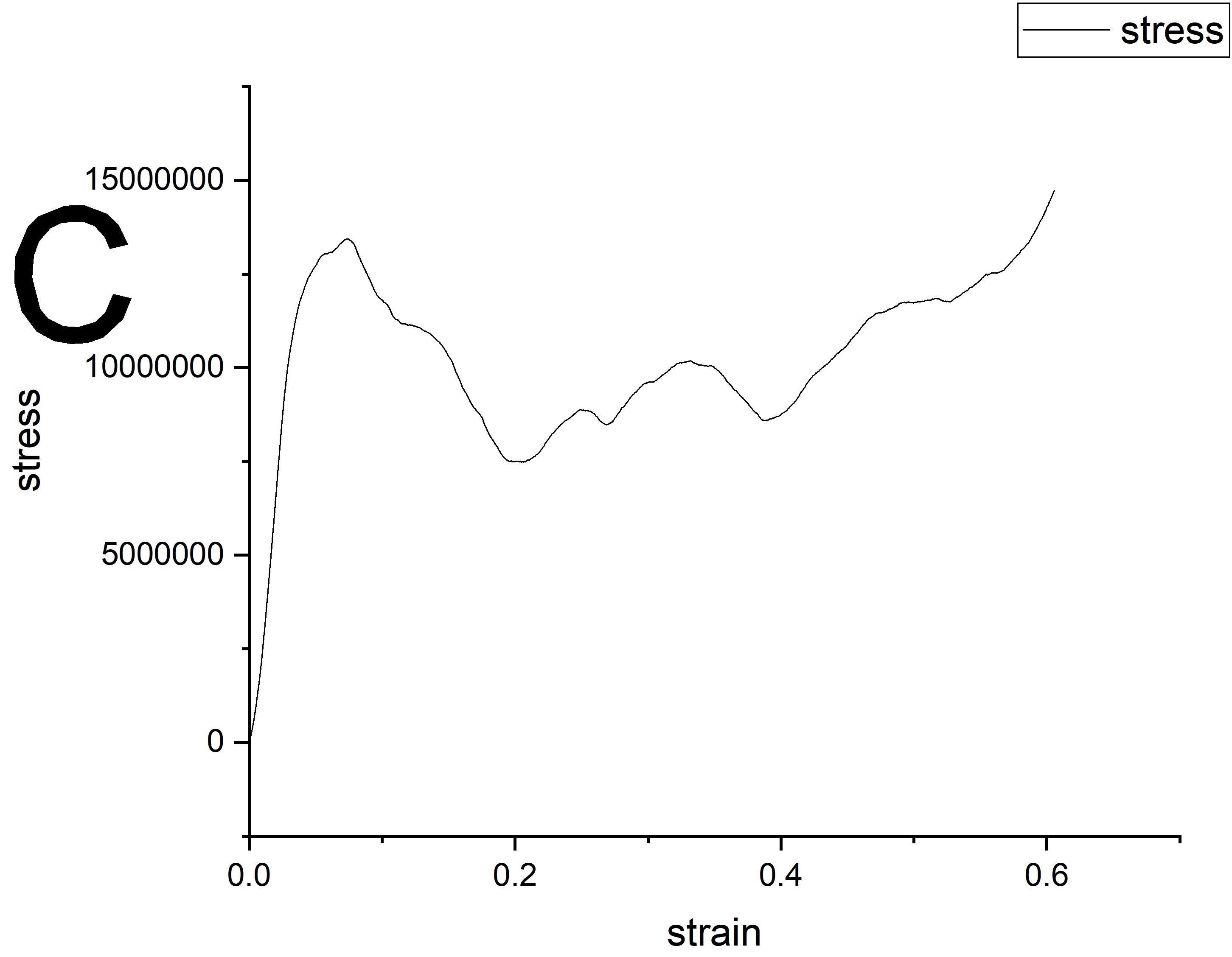

Supplement: Supplementary file 2 — Additional file 2. [file 12891_2022_5888_MOESM2_ESM.zip › sclerosis6R5.tif]
